# Specific Duplication and Dorsoventrally Asymmetric Expression Patterns of *Cycloidea*-Like Genes in Zygomorphic Species of Ranunculaceae

**DOI:** 10.1371/journal.pone.0095727

**Published:** 2014-04-21

**Authors:** Florian Jabbour, Guillaume Cossard, Martine Le Guilloux, Julie Sannier, Sophie Nadot, Catherine Damerval

**Affiliations:** 1 Université Paris-Sud, UMR 0320/UMR 8120, Génétique Végétale, Gif-sur-Yvette, France; 2 Université Paris-Sud, Laboratoire Ecologie, Systématique, Evolution, CNRS UMR 8079, AgroParisTech, Orsay, France; 3 Systematic Botany and Mycology, University of Munich (LMU), Munich, Germany; 4 Muséum National d'Histoire Naturelle, Institut de Systématique, Evolution, Biodiversité, UMR 7205 ISYEB MNHN-CNRS-UPMC-EPHE, Paris, France; 5 Department of Ecology and Evolution, Biophore, University of Lausanne, Lausanne, Switzerland; 6 CNRS, UMR 0320/UMR 8120, Génétique Végétale, Gif-sur-Yvette, France; The University of Tokyo, Japan

## Abstract

Floral bilateral symmetry (zygomorphy) has evolved several times independently in angiosperms from radially symmetrical (actinomorphic) ancestral states. Homologs of the *Antirrhinum majus Cycloidea* gene (*Cyc*) have been shown to control floral symmetry in diverse groups in core eudicots. In the basal eudicot family Ranunculaceae, there is a single evolutionary transition from actinomorphy to zygomorphy in the stem lineage of the tribe Delphinieae. We characterized *Cyc* homologs in 18 genera of Ranunculaceae, including the four genera of Delphinieae, in a sampling that represents the floral morphological diversity of this tribe, and reconstructed the evolutionary history of this gene family in Ranunculaceae. Within each of the two *RanaCyL* (Ranunculaceae *Cycloidea*-like) lineages previously identified, an additional duplication possibly predating the emergence of the Delphinieae was found, resulting in up to four gene copies in zygomorphic species. Expression analyses indicate that the *RanaCyL* paralogs are expressed early in floral buds and that the duration of their expression varies between species and paralog class. At most one *RanaCyL* paralog was expressed during the late stages of floral development in the actinomorphic species studied whereas all paralogs from the zygomorphic species were expressed, composing a species-specific identity code for perianth organs. The contrasted asymmetric patterns of expression observed in the two zygomorphic species is discussed in relation to their distinct perianth architecture.

## Introduction

Bilaterally symmetric flowers are characterized by a single plane of symmetry that separates the flower into two mirror images. The zygomorphic phenotype can be more or less elaborate, involving only organ displacement around the receptacle, or various degrees of morphological differentiation [1], [2]. It is considered an adaptive trait, promoting cross-pollination through optimized interaction between flower shape and pollinator type and behaviour. Comparison of species richness in sister clades that have or have not evolved zygomorphy revealed that bilateral symmetry might have promoted species diversification [3]. Reconstruction of the evolution of floral symmetry states on an angiosperm-wide phylogeny points to more than 70 transitions from an actinomorphic (radially symmetric) ancestral state to zygomorphy [4].

Among angiosperms, the eudicot clade is the most species-rich (about 200,000 species) and is also the one with the highest number of transitions from actinomorphy to zygomorphy [4]. At least 42 of the estimated 46 eudicot-specific transitions have taken place in the derived core eudicot clade. Among basal eudicots, three independent transitions occurred in Ranunculales: one in Ranunculaceae, one in Papaveraceae, and one in Menispermaceae [5]. Ranunculaceae, with more than 2,500 species, is the largest family in the Ranunculales order. This family is especially remarkable by the diversity of its perianth form and architecture, exhibiting variable merism (2- to 5-merism), organ number (determinate or variable), phyllotaxis (spiral or whorled), and perianth differentiation (unipartite or bipartite with inner organs (petals) morphologically differentiated from outer ones (sepals)). The single evolutionary transition from actinomorphy (or pseudo-actinomorphy in the case of spirally inserted organs) to zygomorphy (or pseudo-zygomorphy) took place in the stem lineage of the species-rich Delphinieae tribe (accounting for 26% of all Ranunculaceae species), and has involved the asymmetric formation of spurs in both sepals and petals [5]. The five sepals are spirally inserted, with two ventral, two lateral, and a spurred or hooded dorsal one [6]. The corolla is reduced to four, two, or a single petal, all inserted in the dorsal half of the flower [7]. The other petal primordia stop developing shortly after organogenesis or develop into slender petaloid staminodes [6], [8], [9]. Petal spurs are nectariferous, and their size and shape may play a role in selecting pollinators [10]. Within Ranunculaceae, nectariferous petal spurs also exist in actinomorphic genera, such as *Aquilegia* and *Myosurus*.

Since the pioneering studies demonstrating the key role of *Cycloidea* (*Cyc*) and its paralog *Dichotoma* (*Dich*) in the establishment of bilateral symmetry in *Antirrhinum majus* [11], [12], a number of studies have investigated the evolutionary history of *Cyc*-like genes and their possible role in the evolution of floral symmetry in diverse plant groups. *Cyc* encodes a transcription factor of the plant-specific TCP gene family [13], [14] and is characterized by two typical domains, the TCP and R domains, and an additional ‘ECE’ motif in the inter-domain region [15]. The characterization of homologs of *Cyc* in both monocots and eudicots reveals a complex history of gene duplications [15]–[17]. In the core eudicots, three paralogous lineages have been found: CYC1, CYC2, and CYC3 [15]. All the genes demonstrated or suspected to play a role in bilateral symmetry belong to the CYC2 clade but are not all orthologs due to the independent duplications in various families [18]–[22]. Studies in Poaceae, Zingiberales and Commelinales also suggest a possible role of *Tb*1-like genes, the closest *Cyc* paralog in monocots, in the independent evolution of floral bilateral symmetry [16], [23], [24]. In parallel with the situation observed in core eudicots and monocots, independent duplications of *Cyc*-like genes have been observed in all major lineages of basal eudicots, including the Ranunculales [25]. Studies in Fumarioideae (Papaveraceae) species showed that late expression of the two *Cyc*-like genes was correlated with symmetry type [26], [27]. In the Ranunculaceae, two paralogous lineages have also been found and seem to originate from a duplication event independent from the one that has occurred in the Papaveraceae [25].

In this study, we reconstructed the evolutionary history of *Cyc*-like genes in Ranunculaceae with specific focus on Delphinieae, and compared their expression pattern in actinomorphic and zygomorphic species. We found that the zygomorphic species have generally more gene copies than actinomorphic species. The expression patterns observed in developing flowers varied from species to species, suggesting that the regulation of expression evolved not only after duplication but also after species divergence. Different asymmetric expression patterns were found in the two zygomorphic species studied, which may be related to their different perianth architecture.

## Material and Methods

### Species sampling and plant material

**Figure 1:**
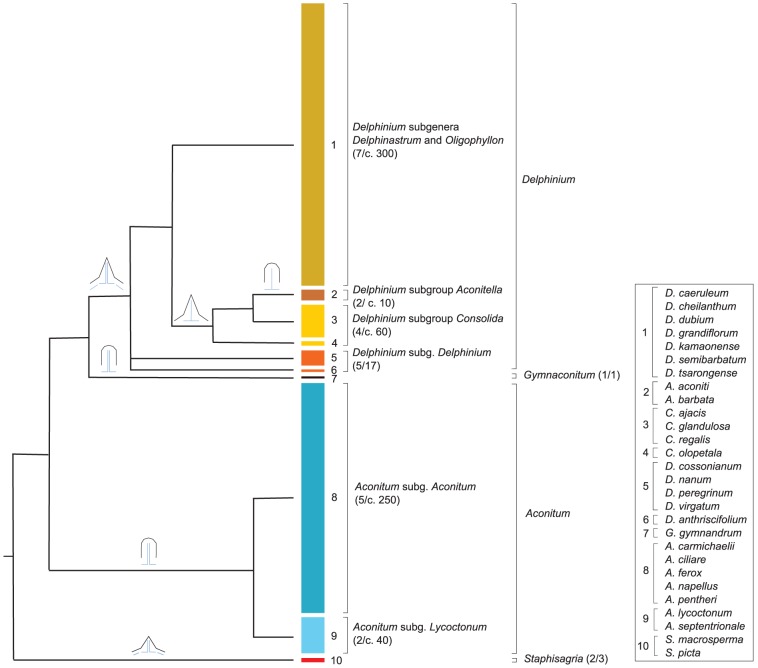
Summary of phylogenetic relationships in the tribe Delphinieae (cf. [7]). The sampling ratio for each of the genera, subgenera, and subgroups from this tribe is shown (the size of each colored rectangle representing the approximate number of species in each). At the bottom right hand corner, the names of the species used for the characterization of *Cyc*-like genes are specified for each of the ten groups. Schematic shapes for the dorsal sepal and corolla are shown above the branches of the tree: the dorsal sepal can be spurred (sharp black shape) or hooded (round black shape); the corolla (in blue) can be composed of four petals (two spurred and two flat), two petals (both spurred), or a single spurred petal.

Forty-nine species (21 actinomorphic and 28 zygomorphic species) representing all subfamilies of Ranunculaceae, except the monotypic Hydrastidoideae (comprising a single genus, *Hydrastis*, which is actinomorphic), were selected for *Cyc*-like gene characterization (Table S1). In particular, all genera, subgenera, and subgroups of Delphinieae, representing the tribe's floral morphological diversity (Table S1, Figure 1), were sampled: the genus *Aconitum* (subgenera *Aconitum* and *Lycoctonum*), the genus *Delphinium* (subgenera *Delphinium*, *Delphinastrum*, *Oligophyllon*, and subgroups *Consolida* and *Aconitella*), the genus *Staphisagria* (recently resurrected by [28]), and the monotypic genus *Gymnaconitum* (recently circumscribed by [29]). The taxonomic divisions within the tribe Delphinieae followed in this paper (except for *Staphisagria* and *Gymnaconitum*) were described in [30].

Material was collected from living plants and herbarium specimens (Table S1). Plants from ten species were grown from seed or were obtained from botanic gardens (the names of the authorities that issued the permit for each location are provided in Table S1) or garden centers, and grown outdoors or in a greenhouse at the UMR de Génétique Végétale (Gif-sur-Yvette. France). Sampling the plants in the wild in France did not concern any endangered or protected species, and did not require any specific permission. Fresh leaf or floral material was harvested and immediately frozen in liquid nitrogen for DNA or RNA extraction, or stored in RNAlater™ (Qiagen) for subsequent RNA extraction.

### Isolation of *Cyc*-like homologs

Genomic DNA was extracted using the NucleoSpin plant kit (Macherey-Nagel, Düren, Germany) for herbarium samples, and following the procedure described in [26] for fresh tissue samples. Characterization of *Cyc* homologs was a two-step process. First, a set of eleven species (six zygomorphic (tribe Delphinieae) and five actinomorphic (subfamilies Ranunculoideae, Thalictroideae, and Glaucidioideae), asterisks in Table S1) was chosen for PCR and cloning effort, and sequence elongation. Nested PCRs were performed on genomic DNA using degenerate oligonucleotides designed in the TCP and R domains (combination III and IV in [26], and additional primers in Table S2). PCR conditions followed [26]. Bands of the expected size (110 to 130 bp for TCP domain amplification, or 300 to 500 bp for the TCP to R amplification) were excised from agarose gel and purified using the QIAquick gel extraction kit (Qiagen). The fragment was ligated into the pGEM-T Easy plasmid (Promega) and transformed into competent subcloning efficiency DH5α *Escherichia coli* cells (Gibco-BRL) following the manufacturer's instructions. Twenty-three to 106 clones were sequenced depending on the species and primer combination. Sequencing was carried out by Genoscreen (Campus de l'Institut Pasteur de Lille, BP245, 59019 Lille, France). Clone sequence alignment distinguished one to three different sequence types (defined on the basis of shared similarities among clones) per species. These sequences were then elongated using inverse PCR. Genomic DNA (∼200 ng) was digested by restriction enzymes without a cutting site within the known sequence in a final volume of 25 µL. Digested DNA (15 µL) was ligated using T4 DNA ligase (1 unit in a final volume of 25 µL). Ligations were heated for 5 minutes at 70°C to stop the reaction, and were purified using the Mini-elute purification Qiagen kit in a final volume of 10 µL. The purified product was used as a matrix for PCR with primers defined in the known sequences. Nested PCRs were performed to increase specificity. PCRs and nested PCRs were performed in 25 µL mix, with 2 µL of purified ligation and 1 µL of first PCR, respectively. Fragments obtained were either directly sequenced or cloned before sequencing, as above.

In a second step, the alignment of the 24 elongated sequences was used to define two sets of three degenerate primers in the TCP and R domains (Cossard *et al.* unpublished and Table S2). These primers were used to perform semi-nested PCRs on genomic DNA from 38 additional species (22 zygomorphic and 16 actinomorphic). PCR products were either sequenced directly or cloned (6 to 19 clones sequenced per species), as described above. Sequences were deposited in GenBank, with accession numbers KJ401946-KJ402052.

### Phylogenetic analyses

Five *Cyc*-like sequences from three species from other Ranunculales families were chosen to root the trees (*Akebia quinata* (Lardizabalaceae): GenBank accession numbers HQ599289-HQ599290, *Circaeaster agrestis* (Circaeasteraceae): HQ599293-HQ599294, and *Nandina domestica* (Berberidaceae) HQ599291). All *Cyc*-like sequences were aligned using MAFFT v. 7.053 [31]; the alignment was visually refined on the basis of amino-acid translation. Highly divergent regions were deleted from the alignment before phylogenetic analyses, resulting in a 381-nucleotide character matrix for global analysis (109 sequences from 48 species). Phylogenetic trees were inferred from these nucleotide alignments using Bayesian inference and maximum likelihood reconstruction methods. Maximum likelihood analyses were performed using raxmlGUI v. 1.3 [32], [33]. Substitution model parameters were estimated using the general time-reversible model with a proportion of invariable sites and a γ-distribution for site-specific rates partitioned by codons (GTR+Γ+I) model. Statistical support for nodes was assessed by bootstrapping the data under the same model (1,000 replicates). Bayesian phylogenetic analyses were carried out using MrBayes v. 3.2.1 [34] using a GTR+Γ+I model. For all three analyses, four chains were run twice for 3,000,000 generations, with a burn-in of 7,500 samples (sample frequency  = 100). Convergence was assessed using the potential scale reduction factor (PSRF = 1.0) and the average standard deviation of split frequencies (<0.01). A majority rule consensus tree with posterior probabilities of nodes was built.

### Molecular evolution studies

**Figure 2:**
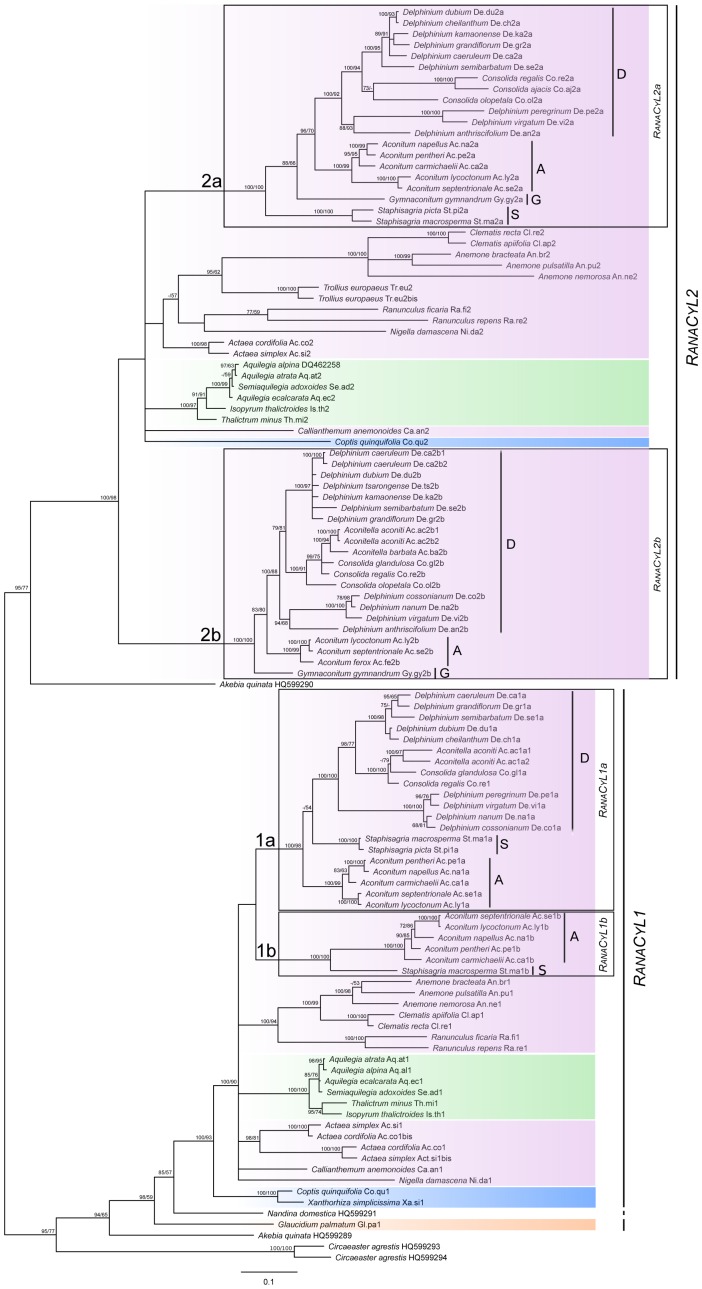
Phylogeny of *Cyc*-like sequences in Ranunculaceae. The phylogeny is inferred using Bayesian and maximum likelihood analysis, rooted in order to group together all the *RanaCyL1* sequences in one clade, and all the *RanaCyL2* sequences in another clade. The tree topology obtained by Bayesian inference is shown. Bayesian posterior probability ≥70 and bootstrap support from maximum likelihood analysis ≥50 are given (hyphen if inferior to those thresholds). The 381-position alignment was generated from 109 *RanaCyL* (from 48 species including 27 Delphinieae) and five other Ranunculales *Cyc*-like sequences (from three species). Portions of the tree are highlighted with colors according to the subfamilies of Ranunculaceae. The species sequenced for *RanaCyL* belong to: pink: Ranunculoideae, green: Thalictroideae, purple: Coptidoideae, and orange: Glaucidioideae. A: *Aconitum*, D: *Delphinium*, G: *Gymnaconitum*, S: *Staphisagria*. The *RanaCyL* branches tested in the molecular evolution analyses are 1a and 1b in *RanaCyL1*, and 2a and 2b in *RanaCyL2*.

We looked for particular selection regimes on the branches subtending the *RanaCyL1* and *RanaCyL2* clades of Delphinieae (see Figure 2 and Figure S1). For each paralog, we used sequences with a minimum of missing data in the TCP and R domains (respectively a 381-position matrix of 44 sequences for RanaCyL1, and a 357-position matrix of 49 sequences for *RanaCyL2*) to reconstruct phylogenetic trees. Maximum likelihood analyses were performed using the online version of PhyML v 3.0 [35], with a GTR+Γ+I evolutionary model; tree improvement was carried out by Subtree Pruning and Regrafting and Nearest-Neighbor Interchange, and branch support evaluated by approximate likelihood-ratio test (SH-like). We then performed tests on ω, calculated as the ratio of the non-synonymous (dN) over the synonymous (dS) substitution rates, using PAML v. 4.4 [36]. We first determined the most parsimonious model of codon frequency that fits the data using the Codonfreq option implemented in the codeml package under a unique ω model. To do so, we compared nested models (equal, F1×4, F3×4, F61) using likelihood ratio tests (LRTs), and retained the “equal” model for all subsequent analyses. Then, using the codeml package, we estimated ω in the branch of interest (foreground branch) and other branches in the tree (background branches). We tested this model against a null hypothesis in which a single ω value was estimated for all branches (model M0), using a hierarchical LRT with a single degree of freedom (df). We also used the Branch-Site model MA with four classes of sites (two classes allowing ω>1 specifically in the foreground branch), that we tested against MA0 (the two foreground specific classes are considered to evolve neutrally), and against model M1a that considers two classes of sites in all branches (one with 0<ω<1, the other with ω1 = 1). Testing model MA against MA0 allows detection of positive selection at some sites in the foreground branch using an LRT with 1 df. Comparing M1a and MA0 reveals relaxed constraints on the foreground branch (LRT with 1 df); the comparison between M1a and MA is a test for MA significance, but it confounds positive selection and relaxed constraints (LRT with 2 df [37]).

### Expression studies

Expression of the *RanaCyL* genes was investigated by semi-quantitative RT-PCR in the leaves, floral buds, and floral dissections of four species: *Aquilegia alpina*, *Nigella damascena* (both actinomorphic), *Aconitum carmichaelii*, and *Consolida regalis* (both zygomorphic). In *A. alpina* and *N. damascena*, three bud sizes were sampled: <1 mm, 2 mm and 3–5 mm. In *A. carmichaelii* and *C. regalis*, buds were harvested at only two stages (<2 mm and 3–5 mm) because of limited material availability. Floral buds at advanced developmental stages were dissected in the four species in order to separate floral organs according to their dorsal/ventral position relatively to the inflorescence axis. For *A. alpina*, *A. carmichaelii*, and *C. regalis*, we could only dissect floral buds from 6–7 mm length. In *A. carmichaelii*, it was not possible to obtain sufficient material for the two dorsal petals that are tiny at this stage. Both 3–5 and 6–7 mm diameter buds were dissected in *N. damascena*. In all four species, calyx phyllotaxis is spiral, and sepal aestivation is quincuncial (imbricate) with two sepals exterior, two interior, and one with one margin interior and the other exterior [38]. The dorsal sepal is the second one in the initiation sequence [6], [39]; it was identified as the second most developed sepal, i.e. the youngest of the two exterior sepals. *Aquilegia alpina* has five deeply spurred petals alternating with the sepals, and *N. damascena* has five to ten (most generally eight) saccate petals. In the dissections of both actinomorphic species, we separated the perianth organs into the dorsal sepal/petal versus the other sepals/petals. The two zygomorphic species have a reduced corolla, with either only two petals (*A. carmichaelii*) or a single petal (*C. regalis*), each of these petals being spurred. This reduced corolla is located in the dorsal part of the flower, and is nested within the dorsal sepal (hooded in *A. carmichaelii*, spurred in *C. regalis*). The perianth organs were separated according to their dorsal, lateral, or ventral position.

Total RNA was extracted using the RNeasy Plant Mini Kit (Qiagen) and processed as described in [26]. PCRs for *actin* were carried out for all samples without RT (and additionally for the *RanaCyL* genes for samples where sufficient material was available) in order to check for absence of DNA contamination. *Actin* was used as a loading control between samples, and amplified under the following conditions: 3 min at 95°C, then 25 cycles of 95°C for 30 s, 52°C for 30 s, 72°C for 2 min, and final elongation at 72°C for 8 min [26]. *RanaCyL* genes were amplified using specific oligonucleotides (Table S2) under the same conditions as *actin* except for the annealing temperature (52°C for *AaCyL1*, 54°C for *NdCyL2*, 56°C for *CrCyL2*a, 58°C for the other genes) and number of cycles (30 for *AaCyL1*, *AaCyL2*, and *AcCyL1*a, 33 cycles for the other genes). Two to three biological replicates and at least two technical replicates were done for each species to validate the reproducibility of the results.

## Results

### Evolutionary history of *Cyc*-like genes in Ranunculaceae

One hundred and nine sequences homologous to *Cyc* were found in the 49 species analyzed; no sequences homologous to *Cyc* were obtained from *Aconitum ciliare*. Most sequences contained the conserved ECE motif between the TCP and R domains. The number of copies was generally higher in Delphinieae than in the other species, with up to four sequences in *Delphinium caeruleum*, *Aconitella aconiti* and *Aconitum septentrionale*.

Maximum likelihood analysis and Bayesian inference were used to reconstruct the evolutionary history of *Cyc*-like genes in the family Ranunculaceae. Both methods produced similar topologies, revealing two well-supported large clades each including one or more non-Ranunculaceae sequences, suggesting a duplication event predating the divergence of Lardizabalaceae and Ranunculaceae (Figure 2). Within both clades (hereafter *RanaCyL1* and *RanaCyL2*), sequence relationship was broadly congruent with species phylogeny at the tribal level, however deeper nodes were poorly supported. Separate analyses were carried out for *RanaCyL1* (48 sequences, 39 species, 369 characters) and *RanaCyL2* (61 sequences, 45 species, 363 characters) without improving the resolution (results not shown). In both *RanaCyL1* and *RanaCyL2*, the Delphinieae sequences formed two well-supported clades (respectively *RanaCyL1*a and b, and *RanaCyL2*a and b) whose relationship with each other and with other sequences was not resolved. Within Delphinieae, the relationship among the *RanaCyL* sequences was congruent with species phylogeny (Figures 1 and 2): *Staphisagria* *Cyc*-like sequences were the earliest-diverging ones (except in the *RanaCyL1*a lineage where their position within the clade is not well supported), *G. gymnandrum* *Cyc*-like sequences and the rest of *Aconitum* *Cyc*-like sequences formed two independent lineages, and *Consolida* and *Aconitella* *Cyc*-like sequences were nested within the *Delphinium* sequences.

### Patterns of molecular evolution in Delphinieae *RanaCyL* sequences

The higher number of *RanaCyL* paralogs found in Delphinieae compared to other Ranunculaceae could suggest that some of these gene lineages are subject to particular evolutionary forces. To test this hypothesis, we investigated patterns of selection at the molecular level in each *RanaCyL* paralog independently, focusing on the branches subtending the Delphinieae *RanaCyL* clades (see Figure 2). For *RanaCyL1*, the branch model suggested higher levels of constraint on the *RanaCyL1*a branch ω_0_ = 0.305, ω_1_ = 0.088, p = 0.0046). None of the Branch-Site models were significant, indicating neither positive selection nor relaxed constraints at specific sites in either branch subtending *RanaCyL1*a or 1b. For *RanaCyL2*, none of the Branch or Branch-Site models were significant, indicating no specific evolutionary pressures on the *RanaCyL2*a or 2b branches compared to other branches in the phylogeny (Table 1).

**Table 1:** Results of the tests of molecular evolution in each sublineage of *RanaCyL1* and *RanaCyL2*.

***RanaCyL1***		
Model	Loglikelihood	Significance of LRT and model parameters
*Null model* M0	−5392.85	ω = 0.296
*Branch*		
ω0 = ω1, ω2	−5392.84	ns
ω0 = ω2, ω1	−5388.83	0.0046 (ω0 = ω2 = 0.305, ω1 = 0.088)
ω0, ω1, ω2	−5388.82	0.0046(ω0 = 0.306, ω2 = 0.287, ω1 = 0.088)
*Site*		
M1a	−5335.13	6×10^−27^, ω = 0.187, p = 0.692
*Branch-Site*		
MA0 (foreground = branch 1a)	−5335.13	ns
MA selection (foreground = branch 1a)	−5335.13	ns
MA0 (foreground = branch 1b)	−5335.13	ns
MA selection (foreground = branch 1b)	−5335.13	ns
***RanaCyL2***		
Model	Loglikelihood	Significance of LRT and model parameters
*Null Model* M0	−5518.37	ω = 0.397
Branch		
ω0 = ω2, ω1	−5518.37	ns
ω0 = ω1, ω2	−5517.85	ns
ω0, ω1, ω2	−5517.84	ns
*Site*		
M1a	−5432.26	2×10^−39^,ω = 0.196, p = 0.528
*Branch-Site*		
MA0 (foreground = branch 2a)	−5432.25	ns
MA selection (foreground = branch 2a)	−5432.18	ns
MA0 (foreground = branch 2b)	−5432.18	ns
MA selection (foreground = branch 2b)	−5431.68	ns

### *RanaCyL* expression during flower development in actinomorphic and zygomorphic species

**Figure 3:**
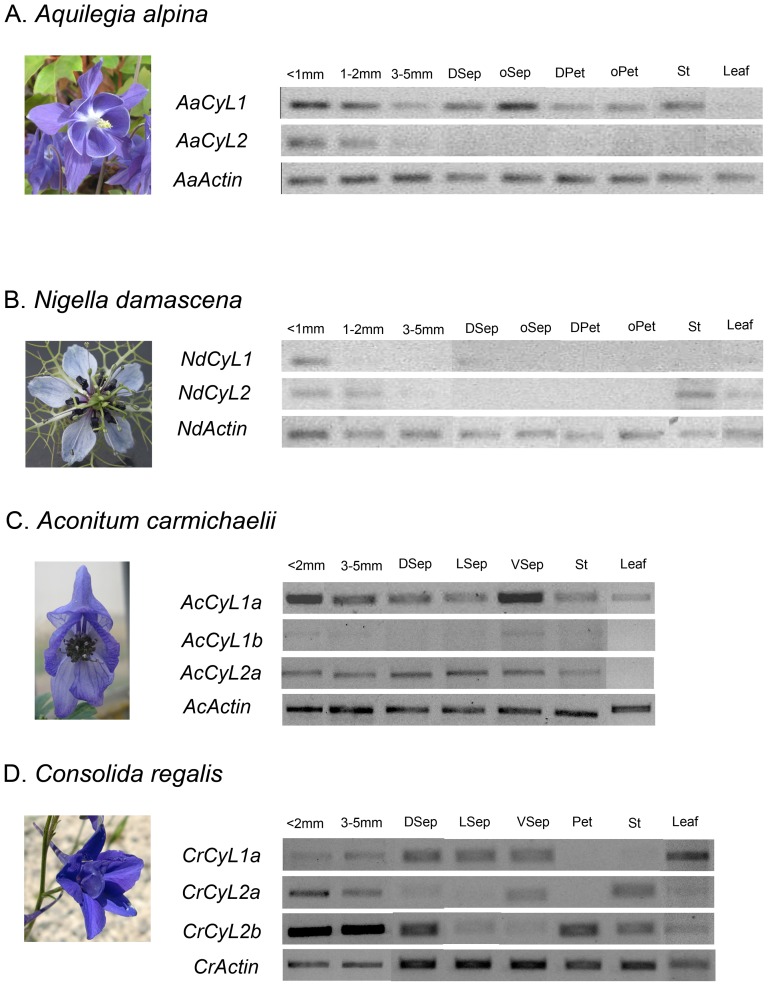
Semi-quantitative RT-PCR analysis of *RanaCyL* gene expression in floral buds, dissected floral organs, and leaves. (A) *Aquilegia alpina*. (B) *Nigella damascena*. (C) *Aconitum carmichaelii*. (D) *Consolida regalis*. For buds, diameter (*Aquilegia* and *Nigella*) or length (*Aconitum* and *Consolida*) are indicated. DSep, oSeP, LSep, VSep: dorsal, non-dorsal, lateral, and ventral sepal, respectively; DPet, oPet: dorsal, non-dorsal petals respectively (actinomorphic species); Pet: spurred petals (zygomorphic species); St: stamens.

Both *RanaCyL1* and *RanaCyL2* genes were found to be expressed in floral buds. Expression was also detected in leaves for three of the four species (Figure 3B, C and D). Their expression level in flowers decreases over time in the actinomorphic species (*AaCyL1*, *AaCyL2*, *NdCyL1, NdCyL2*) but less so in the zygomorphic species (*CrCyL2*a, *AcCyL1a*). In *Aquilegia alpina*, *RanaCyL1* is expressed at a higher level than *RanaCyL2* at the three bud stages whereas it is the opposite in *Nigella damascena* at the last two bud stages (Figure 3A and B). In the zygomorphic species, the signal for *CrCyL2*b appeared to be saturated, and *AcCyL2*a and *CrCyL1*a did not show any difference in the level of expression between the two bud stages investigated (Figure 3C, D). By contrast, duplicates within a same lineage show a differentiated expression pattern: *RanaCyL1*b is very faintly expressed compared to *RanaCyL1*a in *A. carmichaelii*, and *RanaCyL2*b is expressed at higher levels than *RanaCyL2*a in *C. regalis*. In floral dissections of late-stage floral buds, *RanaCyL1* but not *RanaCyL2* was found to be expressed in the perianth organs and stamens of 6–7 mm length buds in *A. alpina*; *AaCyL1* was less expressed in the dorsal sepal than in the set of other sepals (Figure 3A). In 3–5 mm diameter buds of *N. damascena*, *RanaCyL1* is faintly expressed in the dorsal sepal, and *RanaCyL2* expression is restricted to the stamens (Figure 3B); no expression was detected in any organ of 6–7 mm diameter buds (not shown). In *A. carmichaelii*, *RanaCyL1*a expression was detected in all sepals, with higher levels in the ventral ones; a similar expression pattern may exist for *RanaCyL1*b, but this is difficult to ascertain as the gene is very faintly expressed. *RanaCyL2*a expression was detected in all organs examined (Figure 3C). In *Consolida regalis*, *RanaCyL1*a is expressed equally in all sepals but not in petals or stamens. The two *RanaCyL2* paralogs are both expressed in stamens but exhibit a differential expression pattern in the perianth: *RanaCyL2* is more expressed in the dorsal sepal and petal, *RanaCyL2*a is more expressed in the ventral sepals but not in the petal (Figure 3D).

## Discussion

### Lineage-specific duplications of *Cyc*-like genes in Delphinieae

The phylogenetic trees comprising 109 *Cyc*-like sequences from 48 Ranunculaceae species are consistent with the hypothesis of two paralogous lineages originating from a duplication predating the divergence of Lardizabalaceae and Ranunculaceae. These two lineages were previously identified from a small set of Ranunculales species by Citerne *et al.* [25] who suggested that they originated from an early duplication in the order and that the two paralogous lineages previously described in the Papaveraceae [26], [40] were derived from a family-specific duplication in the *RanaCyL1* clade. Most actinomorphic Ranunculaceae species have one copy of each paralog, with the exception of the two *Actaea* species that underwent a specific duplication in the *RanaCyL1* lineage. The sequences obtained from species of the tribe Delphinieae, characterized by zygomorphic flowers, clustered in two well-supported clades in each *RanaCyL* lineage. However, the lack of resolution at the deeper nodes in the phylogenetic trees leaves open the question of the origin of these duplicated lineages. They may have originated from duplication events having taken place in the stem lineage of the tribe, either independently in *RanaCyL1* and *RanaCyL2*, or through a genome wide duplication. However, no polyploidization event specific to Delphinieae has been reported so far. Alternatively, these duplications might have taken place earlier during the diversification of Ranunculaceae, followed by independent losses in the ancestors of the various tribes except Delphinieae. Differential gene loss probably also took place within each sublineage of Delphinieae, even though we cannot exclude that extensive sequence divergence in these groups resulted in amplification failure with our degenerate primers (perhaps the case for *A. ciliare* where we did not amplify any Cyc-like sequence). However, the absence of a *RanaCyL* paralog is often found at the level of entire sublineages, suggesting a non-artefactual result and a non-random genomic event. For instance, while copies from the four *RanaCyL* subclades were present in *Aconitum*, no copy of *RanaCyL1*b was found in *Delphinium*, suggesting gene loss in the ancestor of the genus. Similarly, no copy of *RanaCyL2*b was found in *Staphisagria*, no copy of *RanaCyL2*a was found in the *Delphinium* subgroup *Aconitella*, and no copies of *RanaCyL1* were found in *Gymnaconitum*.

In Dipsacales and Malpighiales (core eudicots), duplications in the CYC2 lineage have coincided with the evolution of zygomorphy [41], [42]. Various studies suggest that in core eudicots, the CYC2 clade compared with the CYC3 and especially the CYC1 clade has undergone repeated duplication events and/or is prone to paralog retention [15], [43], [44]. Classical models predict that following gene duplication, duplicates experience functional divergence through neofunctionalization or subfunctionalization, or accumulate deleterious mutations and degenerate [45], [46]. These processes theoretically leave different evolutionary signatures in gene sequences, but it remains difficult in practice to tease apart the first two models [37], [47]. In sunflower, five Cyc2 genes have been found. Positive selection at four sites scattered in the TCP and R domains may have promoted functional divergence among three of these duplicates, with one paralog exhibiting a ray flower-specific expression whereas the others have wider expression in the inflorescence [48]. In *Lupinus*, a shift in floral architecture has been found to coincide with positive selection operating at a few sites in one CYC2 gene (*LegCyc1B*), suggesting neofunctionalization [49]. Our analyses failed to reveal any signature of positive selection in the Delphinieae sublineages, either in *RanaCyL1* or *RanaCyl2*. By contrast, we observed that the *RanaCyL1*a sublineage experienced a high level of purifying selection compared to other *RanaCyL1* or even *RanaCyL2* genes, suggesting a higher level of functional constraint on this gene, which is consistent with its presence in almost all sublineages of Delphinieae (except in the monotypic genus *Gymnaconitum*). This is similar to what has been described in Antirrhineae, where the Cyc lineage seems to experience a higher level of constraint than the Dich lineage [50]. In addition to a divergence in protein sequence, paralogs that are maintained through evolution generally acquire divergent expression patterns. In the case of transcription factors susceptible to heterodimerize such as TCP proteins, the novel organ and/or developmental stage specific combination of proteins resulting from these regulatory changes may promote the emergence of novel phenotypes by the recruitment of new sets of target genes [44], [51], [52] even in the absence of selection for amino acid replacement.

### Diversity of perianth architecture and expression patterns of *Cyc*-like genes

In Ranunculaceae, the ancestral state for floral symmetry is actinomorphy [5]. An interesting finding of our study is that the two actinomorphic species, each one belonging to the two most species-rich tribes of Ranunculaceae, exhibit a different pattern of *RanaCyL* gene expression in perianth organs. In *A. alpina*, *RanaCyL1* is expressed in both sepals and petals, whereas it is faintly expressed only in the dorsal sepal in *N. damascena*. In core eudicot families where radial symmetry is considered to be the ancestral state, no general trend in the pattern of *Cyc*-like gene expression emerges: in Adoxaceae and Elatinaceae *Cyc*-like genes are broadly expressed across the perianth [42], [53], while expression is absent in Centroplacaceae [42], and transitory in *Arabidopsis* (Brassicaceae) [54]. The same absence of general pattern is observed in cases where actinomorphy is derived from an ancestral zygomorphic state [55]–[61]. This suggests that a variety of processes, depending on *Cyc*-like genes or not, can produce a radially symmetric flower. Our results suggest that in Ranunculaceae, the expression of *Cyc*-like genes is dispensable as related to the elaboration of actinomorphy, or that the *Cyc*-dependent processes controlling actinomorphy have diverged since speciation. In *A. alpina*, the asymmetric expression observed in sepals that are spirally initiated is not observed in petals that are initiated in whorls, raising the hypothesis of a link between *Cyc*-like expression and phyllotaxis.

**Figure 4:**
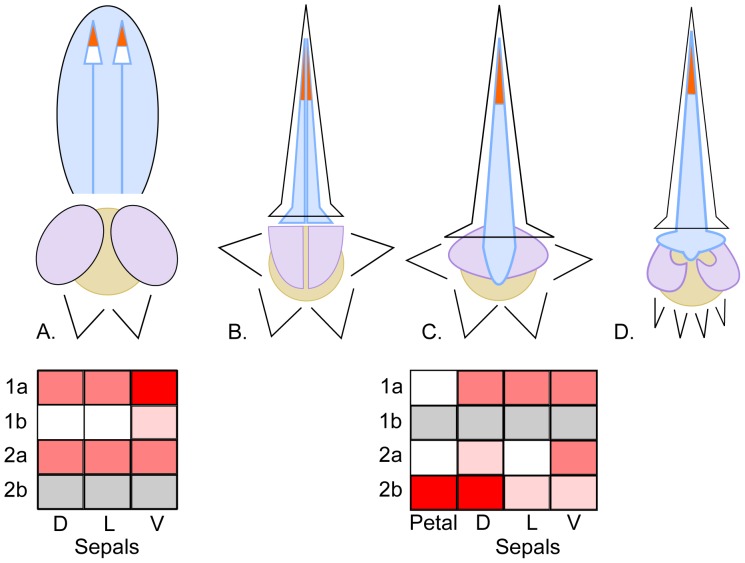
Main types of perianth organization in the tribe Delphinieae. The four sketches are not at the same scale. (A) *Aconitum* type (applicable to groups 7, 8, and 9 from Figure 1). (B) *Delphinium* type (applicable to groups 1, 5, 6, and 10 from Figure 1). (C) *Consolida* type (applicable to groups 3 and 4 from Figure 1). (D) *Aconitella* type (applicable to group 2 from Figure 1). Sepals, dorsal petals, and lateral petals (or lateral lobes of the single petal in *Consolida* and *Aconitella*) are delineated by black, blue, and purple lines, respectively. The organs responsible for covering-protecting the nectar and the sexual organs are colored in light blue and purple, respectively. Nectar is shown in orange; it is secreted and concealed in the spur of the dorsal petal(s). Androecium and gynoecium are presented as a beige disk. The limb of the single petal has three lobes (two lateral lobes, in purple, and one upper lobe, in blue) in *Consolida*, and five (two lower lobes, in purple, and three lateral and upper lobes, in blue) in *Aconitella*. The expression level of each of the four *RanaCyL* paralogs is shown in the grids for *Aconitum carmichaelii* and *Consolida regalis*, in the following perianth compartments: petals (results were available only for *C. regalis*), dorsal (D), lateral (L), and ventral (V) sepals. Increasing expression level is shown with increasing levels of red. White: no expression detected. Grey: the paralog could not be amplified. The expression profiles are schematized based on Figure 3.

Consistent with the single evolutionary origin of zygomorphy in Delphinieae, the establishment of bilateral symmetry in this tribe results from similar ontogenetic processes that involve dorsoventralization through asymmetric corolla and spur formation during late developmental stages [6]. These features are superimposed on diverse perianth types that can be categorized according to the number and shape of the organs, and on the identity of the organs responsible for concealing nectar and for covering the sexual organs, thereby constraining the pollinator's movements to maximize pollen load (Figure 4, [7]). The *Aconitum* perianth type characterizes the flowers of the *Aconitum* and *Gymnaconitum* genera. The corolla is reduced to two dorsal petals, and the calyx is built upon a 3+2 pattern: three large sepals (a dorsal hooded sepal concealing the pair of nectariferous spurred petals, and two lateral rounded sepals protecting the sexual organs, see [62]) and two attenuate ventral sepals (Figure 4, and see Figure 1 in [7]). The *Consolida* perianth type is characterized by a pentamerous calyx with sepal limbs similar in shape and size, a spurred dorsal sepal, and a corolla reduced to a single spurred petal (resulting from the fusion of the two dorsal primordia [7]). In contrast with the *Aconitum* perianth type, the functions of nectar and sexual organ protection are achieved by the dorsal petal spur and by the lateral lobes of the petal limb, respectively. The *Delphinium* type is found in the *Delphinium* and *Staphisagria* genera (Figure 1). The corolla is reduced to four petals (two dorsal spurred petals and two lateral flat petals), and the calyx is built upon a 1+4 pattern: a dorsal spurred sepal and four other sepals (two lateral, two ventral). The petal spurs protect the nectar, and the lateral petals cover the sexual organs. The *Aconitella* type is similar to the *Consolida* type, except that the two lateral and two ventral sepals are grouped in a ventral position. This particular organ positioning, together with the nightcap shape of the dorsal spur, makes this flower resemble an *Aconitum* flower (Figure 1). In the *Aconitella* perianth type, the petal spur protects the nectar, and the two lowermost lobes of the petal limb (comprising a total of five lobes) cover the sexual organs (Figure 4).

Dorsoventral differentiation is the most frequent cause of floral zygomorphy in core eudicots. Since the first studies demonstrating the dorsal expression of *Cyc* and *Dich* in *Antirrhinum majus* [11], [12], similar asymmetric expression patterns of *Cyc*-like genes have been reported in zygomorphic flowers from distantly related core eudicots species [4], [53], [63]–[65]. In the two Delphinieae species *A. carmichaelii* and *C. regalis*, the combined expression of the three *RanaCyL* paralogs composes a sepal identity code (Figure 4) that can be related with the calyx dorsoventral differentiation typical of respectively the *Aconitum* and *Consolida* perianth types. In *A. carmichaelii*, the lateral and dorsal sepals have a similar *RanaCyL* code, paralleling the morphological similarities of these organs typical of the *Aconitum* perianth type, and contrasting with the code of the narrower and flatter ventral sepals. By contrast, in *C. regalis* morphological similarities between lateral and ventral sepals is not echoed in the *RanaCyL* code, since each sepal type exhibits its own code. Comparing *A. carmichaelii* and *C. regalis*, the different expression patterns found for a given paralog result in different codes for homologous sepals, possibly related with their distinct perianth architectures. In particular, the dorsal sepal code differs between *C. regalis* and *A. carmichaelii*, suggesting that different morphogenetic processes may control their elaboration. In each species, the paralog without a counterpart (*RanaCyL2* in *A. carmichaelii* and *RanaCyL1* in *C. regalis*) exhibits an overall homogeneous expression amongst perianth organs, while differential expression patterns were observed for the other paralogs. In *C. regalis*, the two *RanaCyL2* paralogs have undergone complementary subfunctionalization, with a predominantly dorsal expression pattern for *RanaCyL2*b and a predominantly ventral expression pattern for *RanaCyL2*a. By contrast, in *A. carmichaelii* both *RanaCyL1* paralogs are overexpressed in the ventral sepals. Up to now, a primarily ventral expression of *Cyc*-like genes has been reported only in the zygomorphic ray flowers of Asteraceae [19], and in monocots in the ventral tepals of *Commelina* species [23] and in the ventral sepal, petals, and staminodial labellum of *Costus* [16]. Our results suggest that changes in the regulation of *RanacyL* paralogs took place independently in the *Aconitum* and *Consolida* lineages. In the Papaveraceae, both *PapCyL* paralogs exhibit a late asymmetric expression along the transverse symmetry plane in zygomorphic Fumarioideae [27]. These results suggest a large flexibility and evolvability in the regulation of *Cyc*-like genes among the Ranunculales.

## Conclusion

Our study suggests that within Ranunculaceae, additional *Cyc*-like gene duplications coincided with the divergence of Delphinieae and the evolution of zygomorphy in Ranunculaceae. The set of paralogs differed between genera and species in this tribe. In addition, expression patterns of *RanacyL1* genes in floral organs varied between the two species of Delphinieae examined here, with differences correlating with their specific perianth architecture. Both observations raise the question of the role of *Cyc*-like genes in the control of floral architecture in Delphinieae, and the possibility that different evolutionary forces act on the paralogous lineages at the generic, subgeneric, or even subgroup level. This may account for the lack of a detectable selection signature on either *RanaCyL1*a/b or *RanaCyL2*a/b stem branches. To test the correlation between expression patterns and perianth type, a larger species sampling is required, with a more precise monitoring of expression levels over developmental stages. Additionally, our full understanding of the evolution of perianth architecture and *RanaCyL* expression in Delphinieae will also depend on our capacity to resolve the phylogenetic relationships within the Ranunculaceae, and especially to identify the sister group of Delphinieae.

## Supporting Information

Figure S1 **Unrooted phylogenetic trees of** ***RanaCyL*** **sequences used for the tests of molecular evolution.** (A) *RanaCyL1* (44 sequences and 381 positions without gaps). (B) *RanaCyL2* (49 sequences and 357 positions without gaps). The backbones were obtained using PhyML v 3.0 and nodes with support below 0.60 (SH test) were collapsed. The branches tested are 1a and 1b in *RanaCyL1*, and 2a and 2b in *RanaCyL2*.
(TIFF)

Table S1 **List of species, voucher, and GenBank accessions for the sequences generated for the present study.** Group: tribe or subfamily within the family Ranunculaceae. Subgroup: genus or subgenus (or subgroup within the genus). Asterisks point to the set of species used for extensive characterization of *Cyc*-like genes. G: plant grown by the authors, H: herbarium specimen, B: plant grown in a botanical garden, W: plant collected in the wild. M and MSB: herbaria codes, Munich, Germany. The De.gr2b sequence was too short to be submitted to GenBank (177 bp). Its nucleotide sequence is: AGACTCTCACTCGAGATCGCTCGTAAGTTCTTTAATCTTCAAGATATGCTTGGGTACGATAAGGCGAGTAAGACGGTCGAGTGGTTGCTGAGGAAGTCAAAGGATGCAATAAATGAGCTCAGCAAAGGGTCCTGTGGTGAGAATAAGAGTGCATCTTCTATTACTGACTGTGATGTG.
(DOC)

Table S2 **List of primers.** a =  degenerate primers used to characterize *Cyc*-like genes, b =  specific primers used for semi-quantitative PCR analysis. F: forward, R: reverse.
(DOC)
