# Supplementary material for: Specific Duplication and Dorsoventrally Asymmetric Expression Patterns of Cycloidea-Like Genes in Zygomorphic Species of Ranunculaceae
Source: PLoS One. 2014 Apr 21;9(4):e95727. doi: 10.1371/journal.pone.0095727 (PMC3994137; doi:10.1371/journal.pone.0095727)
Supplement: Table S1 — List of species, voucher, and GenBank accessions for the sequences generated for the present study. Group: tribe or subfamily within the family Ranunculaceae. Subgroup: genus or subgenus (or subgroup within the genus). Asterisks point to the set of species used for extensive characterization of Cyc-like genes. G: plant grown by the authors, H: herbarium specimen, B: plant grown in a botanical garden, W: plant collected in the wild. M and MSB: herbaria codes, Munich, Germany. The De.gr2b sequence was too short to be submitted to GenBank (177 bp). Its nucleotide sequence is: AGACTCTCACTCGAGATCGCTCGTAAGTTCTTTAATCTTCAAGATATGCTTGGGTACGATAAGGCGAGTAAGACGGTCGAGTGGTTGCTGAGGAAGTCAAAGGATGCAATAAATGAGCTCAGCAAAGGGTCCTGTGGTGAGAATAAGAGTGCATCTTCTATTACTGACTGTGATGTG. (DOC) [file pone.0095727.s002.doc]

**Table S1**. List of species, voucher, and GenBank accessions for the sequences generated in the present study.

| Group | Subgroup | Species | Origin of the sample/ Voucher | *RanaCyL1* | *RanaCyL2* |
| --- | --- | --- | --- | --- | --- |
| Tribe Delphinieae | Genus *Staphisagria* | *S. picta** | B/ JBL-005051 | St.pi1a: KJ402035 | St.pi2a: KJ402015 |
|  |  | *S. macrosperma** | G/ MNHN 02-58 | St.ma1a: KJ402036 St.ma1b: KJ402009 | St.ma2a: KJ402016 |
|  | Genus *Gymnaconitum* | *G. gymnandrum* | H/ Dickoré 9111 (MSB) | - | Gy.gy2a: KJ402017 Gy.gy2b: KJ401993 |
|  | *Aconitum* subgenus *Aconitum* | *A. carmichaelii* | G/ Horticultural, LM | Ac.ca1a: KJ402037 Ac.ca1b: KJ402010 | Ac.ca2a: KJ402018 |
|  |  | *A. ciliare* | H/ Herrmann 238 (M) | - | - |
|  |  | *A. ferox* | H/ Pcelt 29 (M) | - | Ac.fe2b: KJ401994 |
|  |  | *A. napellus** | G/ Horticultural | Ac.na1a: KJ402038 Ac.na1b: KJ402011 | Ac.na2a: KJ402019 |
|  |  | *A. pentheri* | H/ Podlech & Lippert 26275 (M) | Ac.pe1a: KJ402039 Ac.pe1b: KJ402012 | Ac.pe2a: KJ402020 |
|  | *Aconitum* subgenus *Lycoctonum* | *A. lycoctonum* | B/ JBL-005213 | Ac.ly1a: KJ402040 Ac.ly1b: KJ402013 | Ac.ly2a: KJ402021 Ac.ly2b: KJ401995 |
|  |  | *A. septentrionale* | H/ Dietrich 5808 (M) | Ac.se1a: KJ402041 Ac.se1b: KJ402014 | Ac.se2a: KJ402022 Ac.se2b: KJ401996 |
|  | *Delphinium* subgenus *Delphinium* | *D. anthriscifolium* | H/ Podlech 55468 (MSB) | - | De.an2a: KJ402023 De.an2b: KJ401997 |
|  |  | *D. cossonianum* | H/ Podlech 46655 (MSB) | De.co1a: KJ402042 | De.co2b: KJ401998 |
|  |  | *D. nanum* | H/ Zubizaretta 42606 (M) | De.na1a: KJ402043 | De.na2b: KJ401999 |
|  |  | *D. peregrinum* | H/ Franzén *et al.* 870 (M) | De.pe1a : KJ402044 | De.pe2a : KJ402024 |
|  |  | *D. virgatum* | H/ Zarre 53 (MSB) | De.vi1a: KJ402045 | De.vi2a: KJ402025 De.vi2b: KJ402000 |
|  | *Delphinium* subgenera *Oligophyllon* & *Delphinastrum* | *D. cheilanthum** | B/ PBL-007689 | De.ch1a: KJ402046 | De.ch2a: KJ402026 |
|  |  | *D. caeruleum* | H/ Pcelt s.n. (collection date: 1962, M) | De.ca1a: KJ402047 | De.ca2a: KJ402027 De.ca2b1: KJ401991 De.ca2b2: KJ401989 |
|  |  | *D. dubium* | H/ Merxmüller & Wiedmann 285/64 (M) | De.du1a: KJ402048 | De.du2a: KJ402028 De.du2b: KJ402001 |
|  |  | *D. grandiflorum** | G/ Horticultural | De.gr1a: KJ402049 | De.gr2a: KJ402029 De.gr2b: see footnote |
|  |  | *D. kamaonense* | H/ Wündisch 303 (MSB) | - | De.ka2a: KJ402030 De.ka2b: KJ402002 |
|  |  | *D. semibarbatum* | H/ Belianina *et al.* 9174 (MSB) | De.se1a: KJ402050 | De.se2a: KJ402031 De.se2b: KJ402003 |
|  |  | *D. tsarongense* | H/ Handel-Mazzetti 7934 (M) | - | De.ts2b: KJ402004 |
|  | *Delphinium* subgroup *Consolida* | *C. ajacis* | H/ Merxmüller 8524 (M) | - | Co.aj2a: KJ402032 |
|  |  | *C. glandulosa* | H/ Nydegger 16741 (M) | Co.gl1a: KJ402051 | Co.gl2b: KJ402005 |
|  |  | *C. olopetala* | H/ Nydegger 43795 (MSB) | - | Co.ol2a: KJ402033 Co.ol2b: KJ402006 |
|  |  | *C. regalis** | G/ Horticultural | Co.re1a: KJ402052 | Co.re2a: KJ402034 Co.re2b: KJ402007 |
|  | *Delphinium* subgroup *Aconitella* | *A. aconiti* | H/ Buttler 20006 (M) | Ac.ac1a1: KJ401988 Ac.ac1a2: KJ401987 | Ac.ac2b1: KJ401992 Ac.ac2b2: KJ401990 |
|  |  | *A. barbata* | H/ Podlech 11320 (M) | - | Ac.ba2b: KJ402008 |
| Other Ranunculoideae |  | *Actaea cordifolia* | B/ JBVP-PFP-1999-VN-1326 | Ac.co1: KJ401967 Ac.co1bis: KJ401965 | Ac.co2: KJ401947 |
|  |  | *Actaea simplex* | B/ PBL-006168 | Ac.si1: KJ401968 Ac.si1bis: KJ401966 | Ac.si2: KJ401948 |
|  |  | *Anemone bracteata* | B/ RBGE-19760898*AFBI25 1216*9 | An.br1: KJ401969 | An.br2: KJ401949 |
|  |  | *Anemone nemorosa* | W/ JS-2008-01 | An.ne1: KJ401970 | An.ne2: KJ401950 |
|  |  | *Anemone pulsatilla* | B/ JBL-005427 | An.pu1: KJ401971 | An.pu2: KJ401951 |
|  |  | *Callianthemum anemonoides** | B/ RBGE-1216*2 19870532*BFBI37 | Ca.an1: KJ401972 | Ca.an2: KJ401952 |
|  |  | *Clematis apiifolia* | B/ PBL-015240 | Cl.ap1: KJ401973 | Cl.ap2: KJ401953 |
|  |  | *Clematis recta** | G/ Horticultural | Cl.re1: KJ401974 | Cl.re2: KJ401954 |
|  |  | *Nigella damascena** | G/ MNHN 04-98 | Ni.da1: KJ401975 | Ni.da2: KJ401955 |
|  |  | *Ranunculus ficaria* | W/ JS-2008-03 | Ra.fi1: KJ401976 | Ra.fi2: KJ401956 |
|  |  | *Ranunculus repens* | W/ JS-2008-07 | Ra.re1: KJ401977 | Ra.re2: KJ401957 |
|  |  | *Trollius europeus* | B/ JBL-007382 | - | Tr.eu2: KJ401958 Tr.eu2bis: KJ401946 |
| Subfamily Thalictroideae |  | *Aquilegia alpina** | G/ Horticultural | Aq.al1: KJ401978 | See. Fig. 2 |
|  |  | *Aquilegia atrata* | B/ PBL-006436 | Aq.at1: KJ401979 | Aq.at2: KJ401959 |
|  |  | *Aquilegia ecalcarata* | W/ JS-2008-18 | Aq.ec1: KJ401980 | Aq.ec2: KJ401960 |
|  |  | *Isopyrum thalictroides* | B/ JBL-005043 | Is.th1: KJ401981 | Is.th2: KJ401961 |
|  |  | *Semiaquilegia adoxoides* | B/ RBGE-1216*14 19840268*B | Se.ad1: KJ401982 | Se.ad2: KJ401962 |
|  |  | *Thalictrum minus* | B/ PBL-011490 | Th.mi1: KJ401983 | Th.mi2: KJ401963 |
| Subfamily Coptidioideae |  | *Coptis quinquefolia* | G/ Horticultural | Co.qu1: KJ401984 | Co.qu2: KJ401964 |
|  |  | *Xanthorhiza simplicissima* | B/ GIESS-0-U-4468 | Xa.si1: KJ401985 | - |
| Subfamily Glaucidioideae |  | *Glaucidium palmatum** | G/ Horticultural | Gl.pa1: KJ401986 | - |

Group: tribe or subfamily within the family Ranunculaceae. Subgroup: genus or subgenus (or subgroup within the genus). Asterisks point to the set of species used for extensive characterization of *Cyc*-like genes. G: plant grown by the authors, H: herbarium specimen, B: plant grown in a botanical garden (PBL: Parc Botanique de Launay, France; JBL: Jardin Botanique de Lyon, France, RBGE: Royal Botanic Garden Edinburgh, UK; JBVP: Jardin Botanique de la Ville de Paris, France; GIESS: Botanischer Garten Giessen, Germany), W: plant collected in the wild. M and MSB: herbaria codes, Munich, Germany. The De.gr2b sequence was too short to be submitted to GenBank (177 bp). Its nucleotide sequence is: AGACTCTCACTCGAGATCGCTCGTAAGTTCTTTAATCTTCAAGATATGCTTGGGTACGATAAGGCGAGTAAGACGGTCGAGTGGTTGCTGAGGAAGTCAAAGGATGCAATAAATGAGCTCAGCAAAGGGTCCTGTGGTGAGAATAAGAGTGCATCTTCTATTACTGACTGTGATGTG.
