# Supplementary material for: Specific Duplication and Dorsoventrally Asymmetric Expression Patterns of Cycloidea-Like Genes in Zygomorphic Species of Ranunculaceae
Source: PLoS One. 2014 Apr 21;9(4):e95727. doi: 10.1371/journal.pone.0095727 (PMC3994137; doi:10.1371/journal.pone.0095727)
Supplement: Table S2 — List of primers. a = degenerate primers used to characterize Cyc-like genes, b = specific primers used for semi-quantitative PCR analysis. F: forward, R: reverse. (DOC) [file pone.0095727.s003.doc]

**Table S2.** List of primers.

| Primer name | Use | Sequence 5’ 🡪 3’ |
| --- | --- | --- |
| F1 | a | AARAARGAYCGNCAYAGCAA |
| F2 | a | AGCAARATHNRMACAGCWCAAGG |
| F3 | a | ACAGCWCAAGGYCYWAGRGA |
| F4 | a | ACWGCWCAAGGYAYTMGDGA |
| F4b | a | AAMGAYCGNCAYAGCAARAT |
| F37 | a | GTCCNAGRGAYCGNAGRATG |
| F28 | a | ACWGCNCARGGYAYTMGNGA |
| F5 | a | TTCTTTATGGTTCAAGAYATGC |
| F6 | a | GCWAGYAAAACNGTNGAATGG |
| R1 | a | TCYTKGATRGCDGHYTTBGAC |
| R2 | a | ARCCANTSNASNGTTTTRCT |
| R3 | a | CTTYTCMDTWGTTCKYTC |
| R4 | a | CTTCTCCTTAGTTCKYTC |
| R5 | a | ARCCAWTCNACNGTYTTRC |
| R6 | a | AACCANTCAACAGTYTTRC |
| R7 | a | TAGTYCTCTCCCTTGCCC |
| R8 | a | GATTCCTTTGSAGCGRGTTAC |
| R9 | a | GGATTRAMTGYAGCTTTACG |
| R439 | a | TCYCTNGCTCTTKCTCTTGC |
| R440 | a | TCCCTNGCCCTTNCYCTTG |
| DecCyL1aF | b | TGACAAAGCCAGCAAAACTG |
| DecCyL1aR | b | AAATTCTGCGGGTGATGAAG |
| DecCyL2aF | b | AAAGATCGGCACAGCAAGAT |
| DecCyL2aR | b | CATGCAATTGGGGCTTACTT |
| DecCyL2bF | b | TGAGATCGCCCGTAAGTTCT |
| DecCyL2bR | b | GGGGGTCCACATGTATCAAA |
| AqaCyL1F | b | ACAAGGTCCTAGAGATCGT |
| AqaCyL1R | b | AAGGGGATTGAATGTAGCT |
| AqaCyL2F | b | GCACTAGGGACAGGAGAAT |
| AqaCyL2R | b | GTTGTCGTTTTCTCTCTCA |
| NdCyL1F | b | GAGAATGAGGCTTTCACTT |
| NdCyL1R | b | GAGGATTGAATGCAGCTTT |
| NdCyL2F | b | TTAGGGACCGAAGAATG |
| NdCyL2R | b | GGAGGCAACCACATCTA |
| AccCyL1aF | b | GGAAAAGAACGGACACCAAA |
| AccCyL1aR | b | GGGGGTAGTCAAACATCGAA |
| AccCyL1bF | b | CCCCAATGAACAACGATACC |
| AccCyL1bR | b | AAATCTGGCCACTGCAAGTC |
| AccCyL2aF | b | CTCACATGGAGCATGGAATG |
| AccCyL2aR | b | GCACTGCTTTGATTTCTCCG |

a = degenerate primers used to characterize *Cyc*-like genes, b = specific primers used for semi-quantitative PCR analysis. The specificity was checked by sequencing the amplification product. F: forward, R: reverse.
